# Anabolic effects of IGF-1 signaling on the skeleton

**DOI:** 10.3389/fendo.2013.00006

**Published:** 2013-02-04

**Authors:** Candice G. T. Tahimic, Yongmei Wang, Daniel D. Bikle

**Affiliations:** Endocrine Research Unit, Veterans Affairs Medical Center, Department of Medicine, University of CaliforniaSan Francisco, CA, USA

**Keywords:** IGF-1, osteogenesis, skeletal unloading, mechanotransduction, PTH, integrins, muscle-bone interactions

## Abstract

This review focuses on the anabolic effects of IGF-1 signaling on the skeleton, emphasizing the requirement for IGF-1 signaling in normal bone formation and remodeling. We first discuss the genomic context, splicing variants, and species conservation of the IGF-1 locus. The modulation of IGF-1 action by growth hormone (GH) is then reviewed while also discussing the current model which takes into account the GH-independent actions of IGF-1. Next, the skeletal phenotypes of IGF-1-deficient animals are described in both embryonic and postnatal stages of development, which include severe dwarfism and an undermineralized skeleton. We then highlight two mechanisms by which IGF-1 exerts its anabolic action on the skeleton. Firstly, the role of IGF-1 signaling in the modulation of anabolic effects of parathyroid hormone (PTH) on bone will be discussed, presenting *in vitro* and *in vivo* studies that establish this concept and the proposed underlying molecular mechanisms involving Indian hedgehog (Ihh) and the ephrins. Secondly, the crosstalk of IGF-1 signaling with mechanosensing pathways will be discussed, beginning with the observation that animals subjected to skeletal unloading by hindlimb elevation are unable to mitigate cessation of bone growth despite infusion with IGF-1 and the failure of IGF-1 to activate its receptor in bone marrow stromal cell cultures from unloaded bone. Disrupted crosstalk between IGF-1 signaling and the integrin mechanotransduction pathways is discussed as one of the potential mechanisms for this IGF-1 resistance. Next, emerging paradigms on bone-muscle crosstalk are examined, focusing on the potential role of IGF-1 signaling in modulating such interactions. Finally, we present a future outlook on IGF research.

## OVERVIEW

### GENOMIC CONTEXT AND ALTERNATIVE SPLICING

**FIGURE 1:**
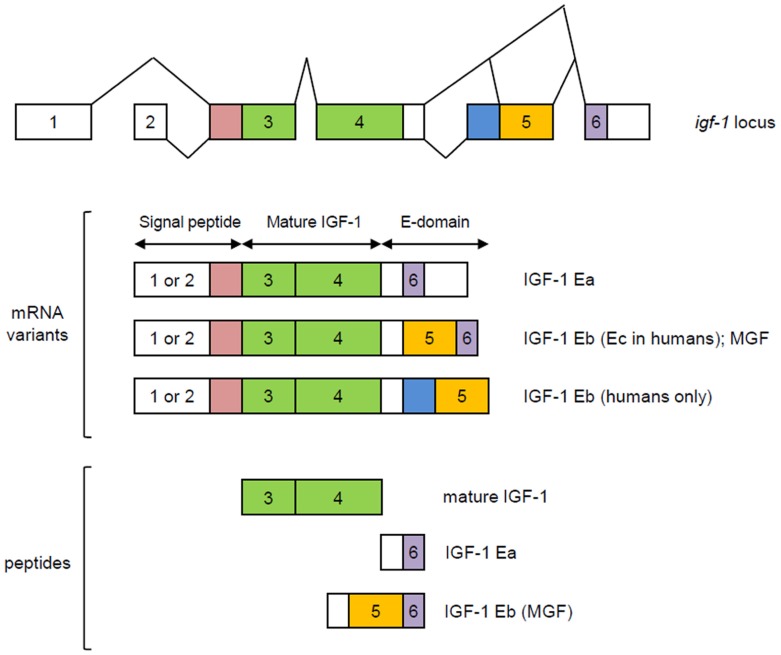
**Splicing variants of the *igf-1* gene**. Variants generated by splicing of exons 4 and exon 6 are referred to as IGF-1Ea while those that contain exons 4, 5, and 6 are designated as IGF-1Eb in rodents and IGF-1Ec in humans. Exons 1 and 2 are used interchangeably and together with the early part of exon 3, code for a signal peptide. Mature IGF-1 is encoded by exons 3 and 4. MGF is generated from exons 5 and 6. Modified from Matheny et al. (2010).

The *igf-1* locus spans about 90 kb of chromosomal DNA. In humans, it is located in chromosome 12 while in mouse it is found in chromosome 10. In both species, nearby neighbors include PAH (phenylalanine hydroxylase), PARPB (PARP1 binding protein), and PMCH (pro-melanin-concentrating hormone). The *igf-1* gene contains six exons, with alternative splicing giving rise to multiple mRNA variants that differ based on the presence of an alternative leader sequence that code for a signal peptide and C-terminal exons (Yang et al., 1995; Barton, 2006; Matheny et al., 2010). In both humans and rodents, variants generated by splicing of exons 4 and exon 6 are referred to as IGF-1Ea while those that contain exons 4, 5, and 6 are designated as IGF-1Eb in rodents and IGF-1Ec in humans (Bell et al., 1986; Rotwein et al., 1986; Chew et al., 1995; **Figure 1**). These splicing variants generate C-terminal extensions called E-domains and are named as such to denote their location relative to the BCAD domains of the mature IGF-1 peptide (Matheny et al., 2010). All currently known splicing variants contain exons 3 and 4 that encode the mature IGF-1 peptide sequence. Translational processing of these variants appear to be complex, giving rise to pre-pro-IGF-1 peptides (Clemmons and Shaw, 1986; Rotwein et al., 1987; Bach et al., 1990), which are cleaved to generate mature IGF-1 and E-domain derivatives referred to as the E-peptides. Mature IGF-1 is a 70 amino acid peptide with a high degree of sequence conservation among mammals (LeRoith et al., 1993; Upton et al., 1998). The IGF-1Eb (IGF-1Ec in humans) variant has been the source of much interest as it has been shown to be up-regulated in muscles subjected to mechanical stimulation (Yang et al., 1996, 1997; McKoy et al., 1999). Hence, the E-peptide generated from this transcript has been separately referred to as mechano-growth factor (MGF).

Although it has been shown that bone displays an IGF-1 isoform profile different from that found in other tissues such as liver (West et al., 1996), it remains unclear how differential expression of these IGF-I isoforms plays a role in osteogenic processes. One of the few studies on this topic has shown that the relative expression of isoforms Ea and Eb remains unchanged during osteoblast differentiation in mice (Smith et al., 2013). However, the authors of the study noted that differentiating osteoblasts from C57BL/6J animals exhibited a preference for the longer of the two 3′ untranslated region (UTR) variants generated from exon 6. Interestingly, no such UTR preferences were observed in the osteoblasts of C3H/He/J animals, which are known to have higher skeletal IGF-1 levels and higher bone mass than C57BL/6J mice. Given that the longer UTR has been shown to be less stable than the shorter variant, this suggests a potential role for certain transcriptional variants of IGF-1 in osteoblast differentiation and the acquisition of peak bone mass. Another study showed that chondrocytes displayed preferential expression of certain isoforms during the course of their differentiation, with Class 1 IGF-1Ea mRNA (uses exon 1 as leader sequence) undergoing up-regulation as chondrocytes switch from a resting to a proliferating state (Lin and Oberbauer, 1999). These two studies associate specific IGF-1 isoforms to key osteogenic events but are limited in the information they provide on the efficiency of these isoforms to promote certain osteogenic processes. To gain more insight on this issue, it will be worthwhile to perform *in vitro* studies to test the capacity of specific isoforms to rescue the phenotypes of osteogenic cells from IGF-1 knockouts.

### IGF-1 AND GH: A HISTORICAL PERSPECTIVE

**FIGURE 2:**
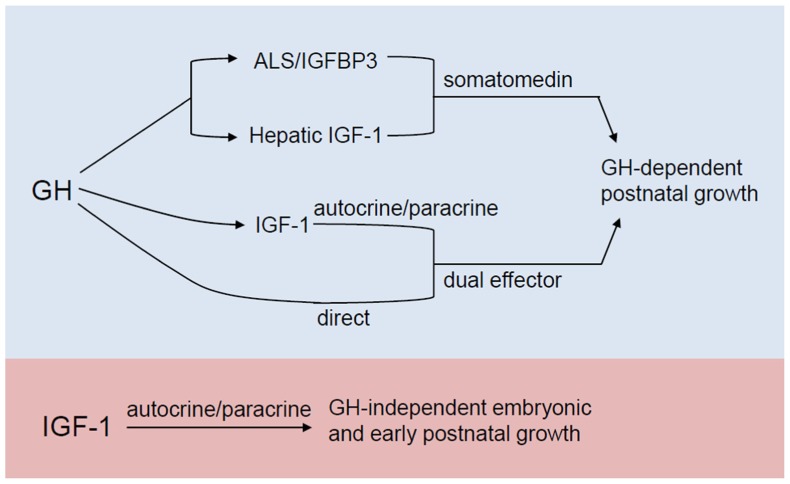
** Revised somatomedin hypothesis as elaborated in Le Roith et al. (2001)**. Apart from its role in stimulating IGF-1 production in the liver, GH also promotes the formation of a ternary IGF binding complex composed of the acid-labile subunit (ALS) and IGFBP-3, which in turn stabilize serum IGF-1. As a dual effector, GH (a) stimulates local IGF-1 production which allows IGF-1 to exert its autocrine/paracrine action on the postnatal skeleton and (b) can also have a direct action on postnatal skeletal growth. In the most current model (blue and purple), the autocrine/paracrine effects of IGF-1 that are independent of GH action during embryonic and early postnatal growth are also accounted for.

Systemic IGF-1 is synthesized primarily in the liver, in a growth hormone (GH)-dependent manner. The somatomedin hypothesis which was first proposed in the early 1970s, provided a model for IGF1 actions on the skeleton (see Le Roith et al., 2001 for review). In its original form, this hypothesis states that growth is determined by the action of pituitary gland-derived GH in the liver, where it stimulates IGF-1 synthesis and release. IGF-1 then circulates to target organs, such as bones and cartilage, acting in an endocrine manner. Circulating IGF-1 then inhibits the further release of GH from the pituitary, completing a negative feedback loop. However, subsequent reports that IGF-1 is also produced in a variety of extrahepatic tissues and can act in an autocrine/paracrine manner (Laviola et al., 2007) necessitated a revised hypothesis called the dual effector theory (Green et al., 1985). It postulates that GH has direct effects on peripheral tissues that are independent of IGF-1, apart from its capacity to stimulate local IGF-1 production in an autocrine/paracrine manner. Succeeding findings again revealed inconsistencies with this revision (reviewed in Le Roith et al., 2001) and that the mechanisms are more complicated than what were initially proposed. The most recent model (**Figure 2**) elaborated in Le Roith et al. (2001) takes into account that apart from its role in stimulating hepatic IGF-1 synthesis, GH also promotes the formation of a ternary IGF binding complex composed of the acid-labile subunit (ALS) and IGF binding protein 3 (IGFBP-3), which in turn act to stabilize serum IGF-1. Moreover, this current model incorporates GH-independent effects of IGF-1 on embryonic growth and reproductive competence, given the findings that *igf-1* global knockouts, as will be discussed in more detail in a later section, show infertility and profound growth retardation at birth, phenotypes that are not observed in GH- or GH receptor-null animals.

### IGF SIGNALING IN THE CONTEXT OF LIGAND-RECEPTOR INTERACTIONS

The IGF system is comprised of type I and type II receptors, their ligands (IGF-1 and IGF-2), IGFBPs, and IGFBP proteases (Le Roith et al., 2001). These proteins primarily mediate stimulation of somatic growth by promoting cell survival, proliferation, and differentiation. Mature IGF-1 and IGF-2 are related peptides of about 8 kDa. The IGFs specifically bind to type I and type II IGF receptors, but due to a 50% homology to insulin, can also interact to a lesser extent with the insulin receptor. The IGFBPs exhibit high affinity binding to both IGF-1 and IGF-2, primarily functioning to modulate the actions of free IGF-1 and IGF-2 (Baxter, 2000). However, IGFBPs may also exert biological effects that are independent of their ability to bind IGFs. The growth-promoting effects of IGFs are primarily mediated via interactions with the type I IGF-1 receptor (IGF-1R). The type II IGF receptor (IGF-2R) on the other hand, is structurally distinct and mainly associates with IGF-2 but can also act as a receptor for ligands that carry mannose-6-phosphate functional groups (Le Roith et al., 2001).

The IGF-1 receptor is a tyrosine protein kinase consisting of two α and two β subunits conjoined by disulfide bridges (Le Roith et al., 2001; Laviola et al., 2007). IGF-1 binding to the cysteine-rich domain of the α subunits leads to sequential phosphorylation of Y1135, Y113, and Y1136 residues of the β subunit which alters the structure of the β chain, thereby turning on tyrosine kinase activity (Favelyukis et al., 2001). These autophosphorylation events and conformational changes create multiple docking sites for a variety of endogenous substrates including members of the insulin receptor substrate (IRS) family which associate with IGF-1R via PTB and SH2 domains, growth receptor binding protein-2 (Grb2) which binds to specific motifs in the IGF-1 receptor as well as in IRS, and the p85 subunit of phosphatidyl inositol-3 kinase (PI3K) which binds to other specific motifs within IRS. Shc, when tyrosine phosphorylated in response to IGF-1, binds to the SH2 domain of Grb2, which in turn forms a complex with Sos, a guanine nucleotide exchange factor. This complex invokes the MAP kinase pathway, resulting in the nuclear translocation of Erk1/2 and subsequent activation of transcription factors such as elk-1 and c-jun. This leads to increased cyclin D1 and down-regulated p21cip and p27kip expression stimulating cell cycle progression from G1 to S phase and thereby completing the pathway by which IGF-1 and other growth factors promote proliferation. Activation of PI3K invokes the Akt pathway, leading to phosphorylation and inactivation of Bad, a proapoptotic member of the Bcl-2 family, in effect blocking apoptosis. However, PI3K and Akt can undergo nuclear translocation and by phosphorylating critical transcription factors also lead to increased cyclin D1 levels.

## IGF-1 IN EMBRYONIC AND POSTNATAL SKELETAL DEVELOPMENT

### CONSEQUENCES OF IGF-1 GLOBAL DEFICIENCY

IGF-1 is a major anabolic signal for both embryonic and postnatal skeletal development. In humans, IGF-1 mutations result in prenatal growth failure that persists postnatally. The skeletal phenotype, as reported in a 15-year old male patient homozygous for a truncation mutation spanning multiple exons of the *igf-1* gene, included a smaller skeleton, severe osteopenia of the lumbar spine and a slight retardation in bone development (Woods et al., 1996). Similarly in the mouse, global knockout of *igf-1* results in a dwarfism phenotype, higher trabecular BV/TV (bone volume per tissue volume), a hypomineralized skeleton and growth plate defects characterized by reduced chondrocyte proliferation and differentiation and increased chondrocyte apoptosis (Baker et al., 1993; Liu et al., 1993; Bikle et al., 2001; Wang et al., 2006b). The consequences of IGF-1 deficiency on skeletal growth become apparent by E13.5, with mutant mice having approximately 90% of the body weight of normal controls. This growth retardation persists throughout gestation such that these animals are born with about 60% of the body weight of their normal littermates. Most of the mutant animals die shortly after birth (Baker et al., 1993; Liu et al., 1993) possibly due to breathing problems caused by an undermineralized ribcage. *Igf-1* null mice that reach adulthood have relative body weights dropping to 32% compared to that of normal animals (Baker et al., 1993). Although the pattern of growth failure seems to be very similar between and *igf-1* null mice and the reported human case of *igf-1* mutation, a number of non-skeletal phenotypic differences do exist. Specifically, the same patient in Woods et al. (1996) was born with a smaller placenta while *igf-1* null mice have placentae of normal size (Baker et al., 1993; Liu et al., 1993; Woods et al., 1996). Moreover, *igf-1* knockout mice have small reproductive organs. This was not observed in the human patient although puberty was slightly delayed (Woods et al., 1996).

Overwhelming evidence from the human and mouse IGF-1 global knockout studies show that IGF-1 plays a critical role in the normal development of the mammalian skeleton (Baker et al., 1993; Powell-Braxton et al., 1993; Bikle et al., 2002; Mohan et al., 2003; Wang et al., 2004, 2006b). As for the mechanism by which IGF-1 regulates skeletal growth and maintenance, it has been proposed that the actions of IGF-1 on bone occur through multiple modes: endocrine, autocrine, and paracrine. Assessments of endocrine actions of IGF-1 on bone were attempted using liver-specific conditional knockouts of IGF-1 (Sjogren et al., 1999, 2002; Yakar et al., 1999). One group, using a Cre recombinase driven by the liver-specific albumin promoter, observed a 75% reduction in serum IGF-1 levels and no defects in skeletal growth of liver-specific *igf-1* mice between ages 1–6 weeks (Yakar et al., 1999). Another group utilized a Cre-lox P system where Cre recombinase was placed under the control of the interferon-responsive Mx promoter and reported similar results using 12-week-old animals (Sjogren et al., 1999). However, a follow-up paper from the same group showed that long-term liver-specific deletion of IGF-1, as assessed in 8–55-week-old mice, leads to small reductions in periosteal bone growth and appendicular skeletal growth with no abnormalities in trabecular bone (Sjogren et al., 2002). The slight but significant effect on periosteal and appendicular skeletal growth could be seen at a much earlier postnatal age (about 2–3 weeks) in mice having a liver-specific double knockout of *igf-1* and the ALS that display a 90% reduction in serum IGF-1 levels. Taken together, these studies suggest that the endocrine action of IGF-1 plays a lesser role in skeletal growth, and is mostly confined to periosteal growth. However, given that liver-specific knockouts of *igf-1* develop subtle growth defects at a much later postnatal age, basal levels of circulating IGF-1 seem to be necessary for the overall process of skeletal growth.

Global IGF-1R knockout embryos exhibit more severe skeletal defects than *igf-1* null animals and die shortly after death, suggesting that both IGF-1 and IGF-2 acting via their shared receptor are involved in regulating skeletal development in the embryonic stage. Since *igf-1r* null animals and *igf-1/igf-1r* double knockouts have similar growth and skeletal defects, it is proposed that the IGF-1R mediates all activities induced by this growth factor (Liu et al., 1993).

### IGF-1 FUNCTION IN OSTEOGENIC CELLS

Because chondrocytes, osteoblasts, osteoclasts, and many other cells in the bone express IGF-1R and IGF-1, global *igf-1* and *igf-1r* knockouts are unable to exclude the effect of systemic perturbations of IGF-1 signaling on the skeleton. A number of studies using conditional knockouts have attempted to overcome such limitations. Studies by our group and others have suggested that locally produced IGF-1 acts in an autocrine/paracrine manner to regulate the proliferation, differentiation, and survival of cell populations that are relevant to the development and maintenance of a healthy skeleton. We shall highlight some of these studies in this section.

#### IGF-1 is important for chondrocyte differentiation

With the exception of the flat bones of the skull and the clavicle, bone formation in the embryo occurs through an endochondral process (refer to Kronenberg, 2003 for review) which begins when mesenchymal stem cells form clusters or condensations via adhesion molecules. Most of the cells in these condensations differentiate into chondrocytes with cells on the periphery forming a perichondrium. Cartilage increases in size through chondrocyte proliferation and secretion of a matrix enriched rich in type II collagen and aggrecan. These chondrocytes become hypertrophic and begin to synthesize type X collagen to induce mineralization of the surrounding matrix while promoting vascular invasion and invoking chondroclasts. Hypertrophic chondrocytes also stimulate nearby perichondrial cells to differentiate into osteoblasts which in turn secrete a characteristic matrix that leads to the formation of a bone collar. Eventually, these hypertrophic chondrocytes are thought to undergo apoptosis (Kronenberg, 2003; Pucci et al., 2007), although this has not been clearly demonstrated, and the alternative hypothesis is that these cells differentiate into osteoblasts.

A major determinant of bone length is the rate of differentiation of proliferating chondrocytes into hypertrophic chondrocytes. The role of IGF-1 in regulating chondrocyte differentiation has been reported in a number of labs including ours (Longobardi et al., 2006; Wang et al., 2006b; Chubinskaya et al., 2008; Zhang et al., 2009), although it must be noted that there are conflicting reports about its role in chondrocyte proliferation (Govoni et al., 2007a; Wang et al., 1999, 2006b). *In vitro*, IGF-1 has been shown to promote chondrocyte proliferation and lengthening of cultured metatarsals (Mushtaq et al., 2004) and to stimulate the production of matrix (Nixon et al., 2001). One group performed chondrocyte-specific ablation of IGF-1 (Govoni et al., 2007a) using a type II collagen I (Col2α1) promoter-driven Cre recombinase (Ovchinnikov et al., 2000) but achieved only a 40% reduction in IGF-1 expression in chondrocytes. No effects on neonatal survival or body size at birth were seen but a 25% reduction in growth and total bone mineral content at 2–4 weeks was noted. Moreover, no statistically significant differences in the proliferation and hypertrophic regions of the growth plate in conditional knockouts versus controls were observed (Govoni et al., 2007a). This differs from our embryonic and postnatal assessments of cartilage-specific knockouts of *igf-1r* which we have generated using the same type II collagen promoter-driven Cre recombinase (^Cart^*igf-1r*, embryonic stage) and a tamoxifen-inducible Cre recombinase (^TamCart^*igf-1r*, postnatal stage). ^Cart^*igf-1r* embryos have shorter, undermineralized skeletons and die shortly after birth. They display growth plate defects such as disorganized chondrocyte columns, delayed ossification, and vascular invasion, decreased cell proliferation, increased apoptosis and delayed chondrocyte maturation. The postnatal effects as evaluated at 2 weeks of age, with 1 week of prior tamoxifen treatment, include growth retardation, disorganized growth plate, and reduced chondrocyte proliferation and differentiation.

A comparison of the skeletal phenotype of global versus cartilage-specific knockouts allows us to better delineate the role of IGF-1 in chondrocytes. The growth plate defects found in the global *igf-1* knockouts are recapitulated in these cartilage-specific *igf-1r* animals, suggesting that IGF-1 is the major ligand acting on this receptor in chondrocytes (Wang et al., 2011). However, it must be noted that even in global *igf-1* knockouts, chondrocyte proliferation is decreased but does not completely stop. Therefore, we propose that the role of IGF-1 in this process lies more on the maintenance of a normal pace of proliferation rather than in the initiation of cell division (Wang et al., 2006b). Moreover, the less severe skeletal defects observed in the ^Cart^*igf-1r* mutants compared to the global knockouts indicates that other cells such as osteoblasts (Zhang et al., 2002), osteoclasts (Wang et al., 2006a), and osteocytes (Reijnders et al., 2007), also contribute to normal skeletal development.

The maturation of chondrocytes from a resting to a hypertrophic state is a critical event in regulating mineralization (Karsenty, 2003). Our work on the *igf-1* knockout mouse indicates a role for IGF-1 in mineralization via regulation of chondrocyte differentiation (Wang et al., 2006b). However, we found this to be site-specific, more severe in the spinal column, ribs, and digits, but limited in its effect in the long bones. In the spinal ossification centers of *igf-1* null animals, the switch from type II collagen to type X collagen expression during chondrocyte differentiation is perturbed as shown by an increased number of type II collagen-expressing prehypertrophic chondrocytes and persistent type II collagen expression in hypertrophic chondrocytes. Moreover, significantly reduced expression of the mineralization marker, osteocalcin was observed, consistent with a failure of chondrocytes to initiate mineralization (Wang et al., 2006b). The similar chondrocyte defects noted in global and cartilage-specific IGF-1R knockout animals indicate that chondrocyte-derived IGF-1 is important for the maintenance of normal chondrocyte maturation. Taken together, the above mentioned studies demonstrate that IGF-1 signaling plays a critical role in the specification of bone size and normal mineralization of the skeleton through the maintenance of normal chondrocyte function.

#### IGF-1 signaling regulates osteoblast maturation and function

A number of *in vitro* and *in vivo* studies have demonstrated the importance of IGF-1 signaling for normal osteoblast development and function. IGF-1 has been shown to stimulate survival (Hill et al., 1997), proliferation, differentiation, and matrix production (Hock et al., 1988) in cultured osteoblast cells. Animals that overexpress an osteocalcin-driven *igf-1* transgene exhibit higher osteocyte lacunae occupancy, increased bone formation rate (BFR), bone volume and bone mineral density (BMD) but without any change in total osteoblasts or osteoclast numbers. These findings suggest that osteoblast-derived IGF-1 primarily exerts it anabolic effects by enhancing osteoblast efficiency and promoting osteocyte survival (Zhao et al., 2000). There is also some evidence for the role of IGF-1 to maintain adequate phosphate uptake by osteoblasts. In human osteoblastic SaOS-2 cells, it has been demonstrated that IGF-1 can promote the transport of inorganic phosphate via the sodium-dependent phosphate transporter, Glvr-1 (Palmer et al., 1997). Moreover, IGF-1 action on osteoblasts has also been shown to promote formation and function of osteoclasts (Hill et al., 1995).

The delayed mineralization observed in global *igf-1* knockouts suggests a role for IGF-1 signaling in normal osteoblast maturation. One group has generated an osteoblast conditional knockout of *igf-1* using a Col1α2 promoter to drive Cre recombinase activity (Govoni et al., 2007b). These animals have a high perinatal mortality and display dwarfism and mineralization defects characteristic of the global *igf-1* knockouts. Reductions in mineral accretion were also noted, and were proposed to result from reduced bone formation at both periosteal and endosteal compartments. Findings from the same study also suggest a slight difference in the degree of requirement for osteoblast-derived IGF-1 in endosteal and periosteal bone compartments, wherein periosteal bone formation tend to be more affected by the loss of IGF-1 in the immature osteoblast. However, data from these conditional knockouts must be interpreted with caution as the authors of the study have pointed out that the Col1α2 promoter-driven Cre recombinase was also highly active in muscle and other non-skeletal tissues (Govoni et al., 2007b). In our own work, mature osteoblast conditional knockouts of *igf-1r* generated via an osteocalcin promoter-driven Cre recombinase have excellent postnatal survival with growth rates that resemble wild-type animals. The Clemens group performed a characterization of the skeletal phenotype of these animals at 3 and 6 weeks (Zhang et al., 2002). Three-week-old conditional knockouts display reduced osteoblast and osteoclast numbers and lower BFR compared to wild-type controls suggesting that IGF-1 signaling is important in promoting osteoblast proliferation and survival during the early stages of postnatal bone modeling. As expected from the coupling of osteoblast-osteoclast functions, the loss of IGF-1 in the osteoblast also negatively affects osteoclast proliferation. However, such differences fail to persist at 6 weeks of age which may potentially reflect compensatory mechanisms. At 6 weeks, notable phenotypes of these animals include an excess of osteoid coupled with reduced mineralization, fewer trabeculae, and lower trabecular BV/TV. No significant change in cortical bone volume was observed in the conditional knockouts compared to controls, possibly due to the fact that the trabecular compartment undergoes the highest rate of remodeling during the time of observation (Zhang et al., 2002). The findings from this study suggest that IGF-1 signaling in the osteoblast is important for osteoblast maturation and the capacity of bone to undergo timely mineralization, findings that we confirmed with *in vitro* studies of osteoblasts from these mice (Wang et al., 2007).

#### Osteocyte-derived IGF-1 is important for normal bone turnover

As osteoblasts mature, they become entrapped in bone matrix to give rise to the osteocyte, the terminally differentiated state of osteogenic cells. The osteocyte is thought to be the primary sensor of mechanical load in bone. Osteocytes form a syncytium, with complex dendritic processes allowing communication of the skeleton’s mechanical loading state to other osteocytes, osteoblasts, and osteoclasts. There is accumulating evidence that the osteocyte plays regulatory roles in bone turnover in response to mechanical load. For example, osteocyte depletion results in reduced BFR, increased osteoclast activity, and a blunted response to skeletal unloading (Tatsumi et al., 2007). Specific deletion of *igf-1* in the osteocyte using dentin matrix acidic phosphoprotein 1 (DMP-1) promoter driven Cre recombinase highlights the role of IGF-1 in regulating bone turnover during developmental bone growth as demonstrated by the reduced serum levels of the bone formation marker, procollagen type I N-terminal propeptide (PINP) and the bone resorption marker, c-telopeptide (CTx) and decreases in osteoclast surface and osteoclast numbers. However, serum levels of IGF-1, calcium, and phosphorus were not affected in these animals, potentially explained by compensatory mechanisms that are in place to guarantee stable serum levels of these critical substances. The skeletal defects of osteocyte specific *igf-1* knockouts recapitulate many of the features of global *igf-1* animals although with less severity. Reductions in total, trabecular, and cortical bone mineral contents were observed in the conditional animals. However, wild-type and conditional animals were indistinguishable with respect to total, trabecular, or cortical bone mineral densities, or in trabecular bone volume, thickness, number, and separation in the secondary spongiosa. Interestingly, osteocyte-specific deletion of *igf-1* also results in the growth plate defects observed in both global and cartilage specific knockouts. Although less severe, the osteocyte-specific *igf-1* knockouts showed a significant reduction in growth plate length that was attributed to the decrease in the length of the hypertrophic zone with no abnormalities in the proliferative zone (Sheng et al., 2013). This suggests a requirement for osteocyte-derived IGF-1 in mediating normal growth plate development, potentially by stimulating chondrocyte maturation. **Table 1** provides a summary of skeletal phenotypes of the various *igf-1* and *igf-1r* knockouts of relevance to bone.

**Table 1:** Summary of skeletal phenotypes of the various *igf-1* and *igf-1r* knockouts.

Target cell	Global	Osteocyte	Osteoblast	Chondrocyte	Hepatic
Gene	*igf-1* (Baker et al., 1993; Liu et al., 1993* Bikle et al., 2001; Wang et al., 2006b ^^^)	*igf-1* (Sheng et al., 2013)	*igf-1* (Govoni et al., 2007b)	*igf-1r* (Zhang et al., 2002)	*igf-1* (Govoni et al., 2007a)	*igf-1r* (Wang et al., 2011)	*igf-1* (Sjogren et al., 1999; Sjogren et al., 2002)
Promoter driving Cre	N.A.	DMP-1	Col1α2	OCN	Col2α1	Col2α1	Mx
Bone length	↓↓	↓	↓	S	S^4d^, ↓4^w^	↓	S<11^w^, ↓>11^w^
Body weight	↓↓	↓	↓↓	S	S4^d^, ↓4^w^	↓	S
Postnatal lethality	+	-	+	-	-	+	-
Serum IGF-1	↓↓	S	S	NR	S	NR	↓↓↓
BMC	↓↓↓	↓	↓↓	NR	↓	NR	S<11^w^, ↓>11^w^
BMD	S	S	↓	NR	S	NR	S
Trabecular BV/TV	↑↑↑	S	S	S3^w^ ↓6^w^	NR	NR	S
Trabecular thickness	↑	S	S	S3^w^ ↓6^w^	NR	NR	S
Trabecular number	↑	S	S	S3^w^ ↓6^w^	NR	NR	S
Trabecular separation	↓	S	S	S3^w^ ↑6^w^	NR	NR	S
Endosteal BFR	NR	↓	↓	NR	NR	NR	NR
Endosteal MAR	NR	↓	↓	NR	NR	NR	NR
Endosteal circumference	NR	↓	↓	NR	↓	NR	NR
Periosteal BFR	↓↓↓	↓↓	↓↓	NR	S	NR	NR
Periosteal MAR	↓↓	↓	↓↓	NR	S	NR	NR
Periosteal circumference	NR	↓	↓	NR	↓	NR	↓^>11w^
Osteoblast number	NR	NR	↓	↓^3w^ S^6w^	NR	NR	NR
Osteoclast number	↓	↓	NR	↓^3w^ S^6w^	NR	NR	NR
Growth plate length	↓	↓	NR	NR	S	↓	NR
Chondrocyte proliferation	S* ↓^^^	S	NR	NR	S	↓	NR
Chondrocyte differentiation	↓	↓	NR	NR	S	↓	NR
Chondrocyte apoptosis	↑	NR	NR	NR	NR	↑	NR
Serum BF markers	NR	↓	NR	NR	NR	NR	S
Serum BR markers	NR	↓↓	NR	NR	NR	NR	S

*BFR, bone formation rate; BMC, bone mineral content; BMD, bone mineral density; MAR, mineral apposition rate; BV/TV, bone volume/total volume; OCN, osteocalcin; 4d, reported at four days; 3w, 4w, 6w, reported at 3, 4, and 6 weeks, respectively; <11w, on or before 11 weeks;>11w, beyond 11 weeks; NA, not applicable, S, no change; NR, not reported. When two studies report opposing phenotypes, a superscript denoting the corresponding citation is indicated.*

## IGF-1 SIGNALING IN THE MODULATION OF ANABOLIC RESPONSES IN BONE

In this section, we shall highlight two mechanisms by which IGF-1 exerts its action on the skeleton, specifically, by modulating the anabolic effects of parathyroid hormone (PTH) and through crosstalk with mechanosensing pathways.

### ROLE OF IGF-1 SIGNALING IN MODULATING PTH ACTION IN BONE

Circulating PTH in its various molecular forms is secreted from the parathyroid glands (D’Amour, 2012). The 84-amino acid mature PTH peptide, referred to as PTH(1-84), exerts most of its biologic actions via 34 amino acids spanning the N-terminal (PTH(1-34)). PTHrP is homologous to PTH in eight of the first amino acids which is sufficient for binding to the same PTH/PTHrP receptor, PTHR1 (Goltzman, 2008). PTH has both anabolic and catabolic actions on bone – when given continuously it is catabolic, when given intermittently it is anabolic. *In vivo*, intermittent administration of PTH leads to a rapid increase in osteocalcin, alkaline phosphatase, and collagen mRNA levels, suggesting its direct effect on osteoblast activity (Dobnig and Turner, 1995; Halloran et al., 1997). Bone marrow stromal cell (BMSC) cultures from animals treated with PTH generate more colony-forming units and a higher percentage of alkaline-positive colonies (Nishida et al., 1994; Kostenuik et al., 1999; Wang et al., 2007), indicating that PTH may also promote proliferation and maturation of osteoblast precursors. Moreover, PTH has been shown to exert anti-apoptotic activity on osteoblasts (Jilka et al., 1999).

Studies from our group and others have demonstrated that IGF-1 is a critical mediator of the anabolic actions of PTH on bone. PTH up-regulates mRNA and protein levels of IGF-1 in bone as assessed *in vivo* (Pfeilschifter et al., 1995; Watson et al., 1995) and *in vitro* (Canalis et al., 1989; Linkhart and Mohan, 1989; McCarthy et al., 1989). In chondrocytes, antibodies to IGF-1 block the ability of PTH and PTHrP to stimulate aggrecan synthesis (Harvey et al., 1999). The skeletal phenotypes of global *igf-1* knockouts have been evaluated for their response to PTH. Five-week-old *igf-1* global knockouts failed to display the anabolic responses to intermittent PTH observed in wild-type animals such as increased serum osteocalcin and alkaline phosphatase levels, increased alkaline phosphatase activity in femoral bone extracts, higher BMD and increased bone formation. In osteoblast cultures from the *igf-1* knockouts, PTH was able to increase cell number only when exogenous IGF-1 was provided (Miyakoshi et al., 2001). In our own assessments, we found that intermittent PTH administration in wild-type mice results in increased fat free body weight, increased cortical thickness and periosteal BFR, increased transcript levels of key osteoblast markers while *igf-1* global knockouts displayed resistance to these anabolic effects (Bikle et al., 2002) as did an osteoblast-specific knockout of *igf-1r*. Moreover, in the osteoblast-specific knockout of *igf-1r*, PTH administration *in vivo* was unable to increase osteoprogenitor number *in vitro* (Wang et al., 2007).

**FIGURE 3:**
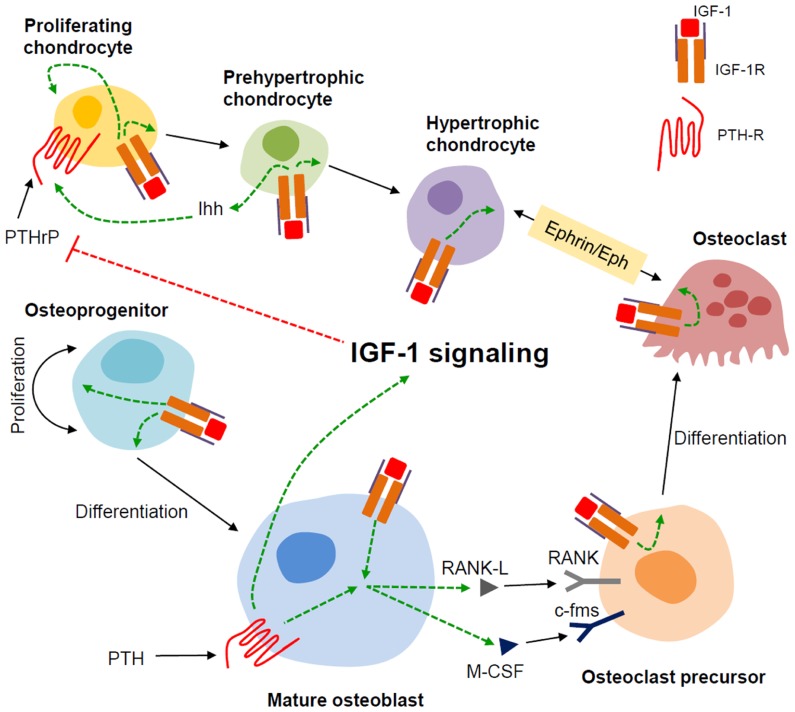
** Model for the role of IGF-1 in modulating the actions of PTH in bone**. We propose that in bone, the mature osteoblast is the major responder to PTH and producer of IGF-1. The IGF-1 induced in the mature osteoblast by PTH stimulates the proliferation and differentiation of osteoprogenitors. IGF-1 thus produced also feeds back on the mature osteoblast to enable PTH to induce RANKL and m-CSF that, along with IGF-1, promote osteoclastogenesis. In chondrocytes, IGF-1 signaling enhances Ihh production in the prehypertrophic chondrocyte. IGF-1 signaling at least in part, inhibits PTHrP production in the proliferating chondrocyte hence stimulating its proliferation and ultimately, its differentiation. IGF-1 signaling also potentiates Ephrin/Eph signaling between hypertrophic chondrocytes and osteoclasts. Dashed green and red lines indicate positive and negative effects, respectively.

The mechanism for the underlying resistance of IGF-1 and IGF-1R-deficient cells to the anabolic effects of PTH is currently the topic of investigation in a number of labs including ours. To this end, we have found that IGF-1 signaling is required for PTH to stimulate RANKL (Wang et al., 2007) and ephrin B2/EphB4 (Wang et al., 2010), see Matsuo and Otaki, 2012 for a review of Ephrins), molecules that potentially affect PTH stimulation of osteoclastogenesis as well as osteoblast proliferation and differentiation. In the chondrocyte, we have proposed a model wherein crosstalk between IGF signaling and the PTH/Indian hedgehog (Ihh) signaling pathways mediate normal chondrocyte maturation. This is based on our observations in the ^Cart^*igf-1r* conditional knockouts, where we found decreased type II collagen and Ihh expression, but increased expression of PTHrP, potentially explaining the growth plate defects in these animals (Wang et al., 2011). The PTHrP/Ihh feedback loop is an autocrine/paracrine pathway (reviewed in Kronenberg, 2006)) that regulates the rate of chondrocyte differentiation (Vortkamp et al., 1996; Karp et al., 2000). In this model, PTHrP is produced by perichondrial and reserve (resting) cells in the embryonic skeleton and diffuses into the proliferation zone to activate PTHR1 in proliferating chondrocytes, thereby sustaining their proliferation and in effect, delaying their maturation (Lee et al., 1995; Chung et al., 1998). As proliferating chondrocytes mature, they produce more Ihh which in turn acts on its receptor Patched (ptch) in nearby cells, resulting in increased PTHrP expression. This cascade of signaling events slows down cell differentiation and prevents premature closure of the growth plate (Karp et al., 2000; Kobayashi et al., 2002). PTHrP is also expressed in prehypertrophic chondrocytes during postnatal growth, consistent with an autocrine/paracrine mechanism for the regulation of chondrocyte maturation at this stage (van der Eerden et al., 2000). In **Figure 3**, we present our working model for the role of IGF-1 signaling in mediating the skeletal actions of PTH in bone.

### IGF-1 SIGNALING AND CROSS TALK WITH MECHANOSENSENSING PATHWAYS

Mechanical loading has anabolic effects on bone leading to increased bone volume and BFR. A classic illustration of this phenomenon is the higher bone mass observed in the playing arm of long-term tennis players versus their non-dominant arm (Haapasalo et al., 1996). On the other hand, skeletal unloading in rodents by hindlimb elevation results in bone loss due to reduced bone formation consequent to impaired osteoblast proliferation and increased apoptosis (Globus et al., 1984; Halloran et al., 1986). A key question to be addressed then, is how the skeleton senses mechanical forces and translates these stimuli into signals that regulate osteogenic proliferation and differentiation. This has relevance in the clinic, as bone lost during prolonged bed rest or immobilization is generally not regained in the elderly, thereby increasing the risk of fractures.

Although the osteocyte is generally considered as the primary sensor of mechanical load, evidence of contributions by other cell populations are present in the literature. In one study, specific ablation of osteocytes using an inducible osteocyte-specific diphtheria toxin gene resulted in increased osteoclast activity, reduced BFR, and a blunted response to skeletal unloading with respect to further increases in osteoclast activity and decrements in BFR. Surprisingly, osteocyte ablation did not block the increase in BFR when the initially unloaded animals were reloaded, indicating that the osteocyte is not the only cell responsible for mechanotransduction (Tatsumi et al., 2007).

Studies from our group and others have revealed a role for IGF-1 signaling in mediating the skeletal response to mechanical load. IGF-1 production and responsiveness are increased in osteocytes and osteoblasts after mechanical load (Lean et al., 1995; Reijnders et al., 2007; Klein-Nulend et al., 2012). Inhibition of IGF-1 with the antagonist IGFBP-4 blunted fluid-flow stress-induced proliferation in the osteoblastic cell line MC3T3-E1 (Kesavan et al., 2011). *In vivo* data support these vitro observations. Transgenic mice overexpressing IGF-1 in osteoblasts exhibit a fivefold increase in periosteal bone formation in response to low magnitude loading. In contrast, there was no significant increase in periosteal bone formation in similarly treated wild-type animals, suggesting that IGF-1 and mechanical load act synergistically to stimulate periosteal bone formation (Gross et al., 2002). The examination of the role of local IGF-1 in mediating the response to mechanical load was undertaken in an osteoblast-specific IGF-1 conditional knockout using type I collagen Cre recombinase. Four point bending resulted in a significant increase in periosteal bone formation in wild-type mice but not in the IGF-1 conditional knockouts, indicating that mechanical loading-induced periosteal bone expansion is dependent on local IGF-1 production in bone. Moreover, axial compression resulted in significant gains in trabecular bone volume, thickness, and density of wild-type mice but not in the conditional knockouts (Kesavan et al., 2011). On the other hand, we have shown that the bone loss of unloading is accompanied by the failure of IGF-1 to increase bone formation and osteoblast proliferation (Sakata et al., 2003). Furthermore, 7-day BMSC cultures from unloaded rats exhibit resistance to the anabolic effects of IGF-1 as shown by failure of IGF-1 to stimulate phosphorylation of its receptor despite unchanged IGF-1R levels and normal binding of IGF-1 to its receptor (Sakata et al., 2004). Concomitant with the resistance to IGF-1, BMSCs from unloaded bone exhibit a decrease in the expression of β1 and β3 integrin subunits (Sakata et al., 2003).

Integrins are membrane-bound single pass receptors that exist as heterodimers composed of an α and β subunit. These proteins, via interactions with their preferred extracellular matrix (ECM), promote cell adhesion while also serving as mechanosensors (reviewed in Harburger and Calderwood, 2009; and Duncan and Turner, 1995). Attachment to the ECM provides a cell with a sense of location and is one of the mechanisms by which a cell perceives deformation of the cell membrane brought about by mechanical stimuli such as shear stress, pressure, or strain. The role of integrins in regulating IGF-1 responsiveness has been demonstrated in a number of studies. One group has shown that αvβ3 integrin expression enhanced IGF-1-induced proliferation of Chinese hamster ovary cells while also providing evidence for a regulatory mechanism that involves ternary complex formation among IGF-1R, IGF-1, and αvβ3 integrin (Saegusa et al., 2009). We have reported that 7-day BMSC cultures from normally loaded animals that have been treated with the disintegrin echistatin or siRNA against β1 and β3 integrins recapitulate the IGF-1 resistance that we observed in BMSCs of unloaded animals (Long et al., 2011). In aortic smooth muscle cells, it has been found that the disintegrin echistatin or blocking antibodies to αvβ3 integrin blocked IGF-1-stimulated proliferation, IGF-1R autophosphorylation, IRS-1 phosphorylation, and binding of the p85 subunit of PI3K to IRS-1. The proposed mechanism in smooth muscle cells is that integrin activation recruits the tyrosine phosphatase SHP-2 to the β3 integrin subunit. IGF-1 in turn, via IGF-1R, phosphorylates and activates the transmembrane protein SHPS-1, which recruits SHP-2 to SHPS-1. When αvβ3 integrin activation is blocked, SHP-2 is instead recruited to IGF-1R where it dephosphorylates and so terminates the activation of IGF-1R (Zheng and Clemmons, 1998; Maile et al., 2001; Clemmons and Maile, 2005). However, our data indicate that bone cells employ a different mechanism for the regulation of IGF-1 responsiveness via integrin signaling (Sakata et al., 2003). We have shown that although echistatin blocks IGF-1 activation of IGF-1R, neither skeletal unloading nor echistatin alters the recruitment of SHP-2 to IGF-1R. Instead, IGF-1R is just not phosphorylated despite the presence of IGF-1 in BMSCs from unloaded bone or normal cells treated with echistatin. Hence, there are at least two distinct mechanisms for regulating IGF-1R activity in response to a cell’s mechanical loading state. In smooth muscle, unloading accelerates the deactivation of the IGF-1R, whereas in bone, skeletal unloading results in failure to activate IGF-1R.

**FIGURE 4:**
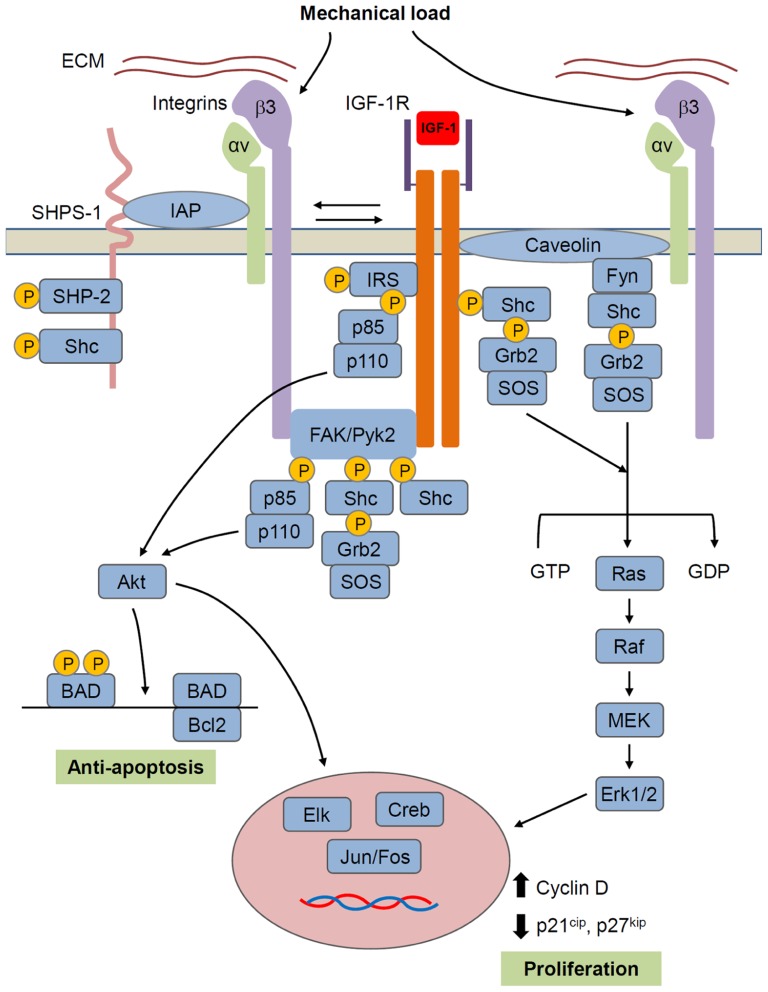
**Model for IGF-1R/integrin interactions in bone cells**. IGF-1R forms a complex with αvβ3 integrin that is required for IGF-1 activation of IGF-1R. Mechanical load increases whereas unloading decreases formation of this complex and thus regulates IGF-1 responsiveness. Formation of the integrin/IGFIR complex brings to IGF-1R, non-receptor kinases such as FAK family and src family kinases that are hypothesized to activate IGF-1R independently and/or synergistically with IGF-1. Caveolin may also contribute to the formation of an integrin/IGF-1R complex. SHPS-1/SHP-2 has been shown to play a role in regulating IGF-1 signaling in some tissues, but this role in bone cells is unclear. IGF-1R signals through two main pathways involving Akt and Ras/Raf/MEK/ERK, respectively. Akt, by phosphorylating BAD, has an anti-apoptotic effect, while ERK promotes proliferation. However, these represent just two of a number of intracellular events triggered by the activated IGF-1R.

Taken together, these studies suggest that growth factor signaling and mechanosensing pathways cooperatively regulate the anabolic response to mechanical load, and that one of the mechanisms by which this is achieved is via crosstalk between the IGF-1 and integrin signaling pathways. Given that IGF-1 and integrin signaling share common downstream effectors such as MAPK and Akt, we propose a model (**Figure 4**) in bone wherein mechanical stimulation results in the activation of both IGF-1R and the integrins. Whether activation of both molecules occur simultaneously or whether one molecule precedes the other is currently unclear. In either case, this leads to amplification of pro-survival (Akt) and proliferation (MAPK) signals ultimately generating an enhanced anabolic response in osteogenic cells. Conversely, the absence of mechanical stimuli suppresses integrin expression and activation which in turn blunts IGF-1 signaling, thereby limiting the anabolic effects of IGF-1 in the skeleton.

## EMERGING PARADIGMS: BONE-MUSCLE INTERACTIONS AND THE POTENTIAL ROLE OF IGF-1 SIGNALING

Bone and muscle are anatomically intimate organs that are associated via tendons and Sharpey’s fibers. Given that muscle contraction places an immense physiological load on bone, the strength of bone must be adapted to muscle strength (Schoenau, 2006). Hence it is thought that muscle and bone grow in proportion to each another. There is some evidence suggesting that IGF signaling plays a role in the coordinated maintenance of a healthy musculoskeletal system. In *igf-1* global knockouts, the osteopenic phenotype is accompanied by muscle hypoplasia attributed to reduced number of cells but not of cell size (Liu et al., 1993). Aside from its anabolic action in bone, IGF-1 is also known to induce muscle cell proliferation and hypertrophy (reviewed in Schoenau, 2006) both *in vitro* and *in vivo* (Adams and McCue, 1998; Chakravarthy et al., 2000; Musarò et al., 2001; Rommel et al., 2001; Vyas et al., 2002). Cultured myotubes treated with IGF-1 undergo hypertrophy and produce more protein (Rommel et al., 2001; Vyas et al., 2002). On the other hand, administration of IGF-1 to rodents results in increased muscle mass (Adams and McCue, 1998; Musarò et al., 2001). It has been shown that the enhanced protein synthesis in muscle occurs through activation of the PI3K-Akt-mTOR signaling pathway (Rommel et al., 2001; Li et al., 2002; Vyas et al., 2002).

Although a detailed characterization of muscle phenotypes has yet to be performed, osteoprogenitor *igf-1r* (^Osterix-Cre^*igf-1r*) and ^Cart^*igf-1r* conditional knockouts, display proportionately smaller muscles concomitant with a shorter and smaller skeleton (unpublished qualitative observations). In the osteoblast *igf-1* knockout using type I collagen Cre, IGF-1 expression was reduced fivefold in muscle (Govoni et al., 2007b). Moreover, the osteocyte-specific knockout of *igf-1*, displays a 59% reduction of IGF-1 mRNA levels in skeletal muscle, although gross muscle architecture appeared normal (Sheng et al., 2013). However, a definitive conclusion on the actual role of IGF-1 in the coordinated maintenance of normal musculoskeletal function has been elusive as most of these promoters have been found to also be active to some degree in muscle. Although the endocrine/paracrine effects of bone on muscle or vice versa cannot be firmly established at present, these findings indicate that muscle and bone share similar IGF-1 responsive gene networks, strengthening the case for the role of IGF signaling in coordinated maintenance of bone and muscle. Research along this theme has very important clinical applications as identification of common signaling mechanisms in muscle and bone may generate therapeutics that simultaneously address the osteopenia and muscle atrophy that is prevalent in the frail elderly.

## CONCLUDING REMARKS: TYING UP LOOSE ENDS IN THE IGF STORY

Although an enormous volume of research spanning at least four decades has provided a substantial understanding of IGF-1 signaling and has even resulted in clinical interventions for growth impairment, much is yet to be learned with regards to its role in the maintenance of a healthy skeleton. Specifically, a better delineation of the embryonic and postnatal roles of IGF-1 signaling in promoting skeletal health is much warranted and will require the use of animal models carrying inducible Cre recombinase transgenes that allow time- and cell-specific deletion of the gene in question. Fortunately, a number of tamoxifen-inducible Cre systems that are of relevance to bone are now available directly from investigators or from the Jax Repository. Our group and a number of others are moving forward in this direction.

To complement these *in vivo* efforts, *in vitro* studies that examine the effects of perturbing ligand-receptor interactions in specific ostegenic cell types will be valuable in providing a more complete understanding of how IGFs elicit their biological effects. Through the years, numerous IGF and insulin analogs have been generated and characterized. However, retrieving and comparing information to find the appropriate analog or to aid in the design of newer molecules is a non-trivial task due to the varying assay formats used by different studies. To address this issue, a group has generated an online public database called IGFmdb, which serves as a centralized source of IGF mutation data in a comparable format with direct links to the original binding studies (Rajapaksha et al., 2012). Their report even provides a case study on how to mine the database for the purpose of designing an experiment to determine the role of IGF-2 acting via the IGF-2 receptor.

The current paradigm for skeletal growth holds IGF-1 as a master regulator for skeletal growth and the acquisition of peak bone mass, and much effort has been invested to understand the anabolic pathways downstream of receptor binding. However, evidence has emerged from studies that focus on the consequences of epigenetic perturbations in development, indicating that the IGF-1/GH axis itself is subject to upstream regulation by the paternally imprinted gene *Rasgrf1* (Itier et al., 1998; Drake et al., 2009). *Rasgrf1* null animals exhibit decreased postnatal skeletal growth, decreased serum IGF-1 levels and reduced IGF-1 transcripts in the liver. Future work focusing on a more thorough evaluation of the skeletal phenotypes of these animals will be of interest to the bone research community.

Finally, a less dismissive stance must be held with regards to the role of IGF-2 in the postnatal skeleton. Although a number of studies suggest that IGF-1 appears to be more relevant to postnatal growth, the role of IGF-2 at this stage of development and its potential relevance to the regeneration of osteogenic stem cells should not be ignored. It has been shown that miR-675, a microRNA product that arises from coordinated epigenetic regulation of the *igf-2/h19* tandem locus (reviewed in Ratajczak et al., 2012) can down-regulate IGF-1R expression (Keniry et al., 2012). Moreover, the *igf-2/h19* tandem locus also through its imprinting status is thought to play a regulatory role in the maintenance of the quiescent state of adult-derived very small embryonic-like stem cells (VSELs; Kucia et al., 2011, 2012). VSELs are thought to be deposited in adult tissues during early embryogenesis to serve as back-up precursor stem cells for more committed cell populations (Ratajczak et al., 2012). Their capacity to form bone in an animal model for skeletal injury has been demonstrated (Havens et al., 2012). These findings open the exciting possibility of manipulating the epigenetic state of the *igf-2/h19* locus to enhance the *ex vivo* expansion of osteogenic populations.

The many unanswered questions that are of high relevance to human health and the diversity of potential research themes afforded by its complex regulatory mechanisms will make the IGF system a rewarding and exciting field of study for many years to come.

## Conflict of Interest Statement

The authors declare that the research was conducted in the absence of any commercial or financial relationships that could be construed as a potential conflict of interest.
